# *POLE* and *POLD1* screening in 155 patients with multiple polyps and early-onset colorectal cancer

**DOI:** 10.18632/oncotarget.15810

**Published:** 2017-03-01

**Authors:** Clara Esteban-Jurado, David Giménez-Zaragoza, Jenifer Muñoz, Sebastià Franch-Expósito, Miriam Álvarez-Barona, Teresa Ocaña, Miriam Cuatrecasas, Sabela Carballal, María López-Cerón, Maria Marti-Solano, Marcos Díaz-Gay, Tom van Wezel, Antoni Castells, Luis Bujanda, Judith Balmaña, Victoria Gonzalo, Gemma Llort, Clara Ruiz-Ponte, Joaquín Cubiella, Francesc Balaguer, Rosa Aligué, Sergi Castellví-Bel

**Affiliations:** ^1^ Gastroenterology Department, Hospital Clínic, Institut d'Investigacions Biomèdiques August Pi i Sunyer (IDIBAPS), Centro de Investigación Biomédica en Red de Enfermedades Hepáticas y Digestivas (CIBEREHD), University of Barcelona, Barcelona, Catalonia, Spain; ^2^ Biomedical Sciences Department, School of Medicine, University de Barcelona, Institut d'Investigacions Biomèdiques August Pi i Sunyer (IDIBAPS), Barcelona, Catalonia, Spain; ^3^ Galician Public Foundation of Genomic Medicine (FPGMX), Centro de Investigación Biomédica en Red de Enfermedades Raras (CIBERER), Genomics Medicine Group, Hospital Clínico, Santiago de Compostela, University of Santiago de Compostela, Galicia, Spain; ^4^ Department of Pathology, Hospital Clinic, Biobanc Clinic-IDIBAPS, Barcelona, Catalonia, Spain; ^5^ Department of Pharmaceutical Chemistry, Philipps-University Marburg, Marburg, Germany; ^6^ Leiden University Medical Center (LUMC), Leiden, Netherlands; ^7^ Gastroenterology Department, Hospital Donostia–Instituto Biodonostia, Centro de Investigación Biomédica en Red de Enfermedades Hepáticas y Digestivas (CIBEREHD), Basque Country University (UPV/EHU), San Sebastián, Spain; ^8^ High Risk and Cancer Prevention Unit, Medical Oncology Department, University Hospital Vall d'Hebron and Vall d'Hebron Institute of Oncology, Barcelona, Spain; ^9^ Gastroenterology Department, Hospital Universitari Mútua de Terrassa, Terrassa, Barcelona, Spain; ^10^ Clinical Oncology Department, Corporacio Parc Tauli, Sabadell, Barcelona, Spain; ^11^ Gastroenterology Department, Complexo Hospitalario Universitario de Ourense, Instituto de Investigación Biomédica Ourense, Pontevedra y Vigo, Ourense, Spain

**Keywords:** colorectal neoplasm, colorectal adenoma, genetic predisposition to disease, POLE, POLD1

## Abstract

Germline mutations in *POLE* and *POLD1* have been shown to cause predisposition to colorectal multiple polyposis and a wide range of neoplasms, early-onset colorectal cancer being the most prevalent. In order to find additional mutations affecting the proofreading activity of these polymerases, we sequenced its exonuclease domain in 155 patients with multiple polyps or an early-onset colorectal cancer phenotype without alterations in the known hereditary colorectal cancer genes. Interestingly, none of the previously reported mutations in *POLE* and *POLD1* were found. On the other hand, among the genetic variants detected, only two of them stood out as putative pathogenic in the *POLE* gene, c.1359 + 46del71 and c.1420G > A (p.Val474Ile). The first variant, detected in two families, was not proven to alter correct RNA splicing. Contrarily, c.1420G > A (p.Val474Ile) was detected in one early-onset colorectal cancer patient and located right next to the exonuclease domain. The pathogenicity of this change was suggested by its rarity and bioinformatics predictions, and it was further indicated by functional assays in *Schizosaccharomyces pombe*. This is the first study to functionally analyze a *POLE* genetic variant outside the exonuclease domain and widens the spectrum of genetic changes in this DNA polymerase that could lead to colorectal cancer predisposition.

## INTRODUCTION

Colorectal cancer (CRC) is one of the most common tumors and an important cause of mortality in the developed world [1]. It is caused by environmental and genetic factors, with 35% of the variation in CRC susceptibility probably explained by inherited causes [2]. The best known examples of inherited CRC predisposition are Mendelian forms such as Lynch syndrome and familial adenomatous polyposis. They account for ~5% of all cases, and are due to germline mutations in *APC*, *MUTYH* and the mismatch repair (MMR) genes, which confer a high risk of developing this disease [3]. However, there is still a considerable number of cases with strong familial CRC aggregation and early disease onset with an unknown inherited genetic basis. An example could correspond to familial CRC type X, where Amsterdam clinical criteria used for Lynch Syndrome identification are fulfilled but there are no alterations in the MMR system [4].

In order to identify new inherited risk factors, whole-genome sequencing combined with linkage disequilibrium studies were recently conducted in families affected by multiple colorectal adenomas and early-onset CRC [5]. By doing so, p.Leu424Val and p.Ser478Asn mutations in *POLE* and *POLD1* DNA polymerases, respectively, were established as a new high-penetrance cause of germline CRC predisposition with an autosomal dominant pattern of inheritance. *POLD1* p.Ser478Asn was also found to be involved in germline predisposition to endometrial cancer [5, 6]. These mutations are located in the exonuclease domain of the protein, which has proofreading activity by removing misincorporated nucleotides during DNA replication [7]. Therefore, mutations in this protein domain will disrupt the fidelity of DNA replication, which will lead to a mutator phenotype, resulting in tumorigenesis. The pathogenicity of these first identified variants was confirmed by functional studies in the orthologous genes in yeast. Regarding somatic studies, tumors from *POLE* and *POLD1* mutation carriers showed a hypermutated phenotype with an excess of G > T/C > A and C > T/G > A transversions, especially in the context TCT > TAT and TCG > TTG [5, 8–10].

Since the discovery of these two new hereditary CRC genes, some additional efforts have been made to characterize the mutational spectrum and the clinical features associated to this new syndrome, which was accordingly named polymerase proofreading-associated polyposis [6]. To this date, *POLE* mutations have been found to be the germline predisposition factor in families with multiple adenomas and early-onset CRC [11–16], as well as in other neoplasms such as endometrial, ovarian, brain, pancreas, small intestine and cutaneous melanoma [15, 17–19]. On the other hand, *POLD1* mutations have been found to predispose carriers to multiple adenomas, CRC, endometrial and breast cancer [11, 14, 15]. Recently, some CRC patients with deficient MMR system caused by tumor biallelic inactivation were reported to also carry germline mutations in *POLE*, these MMR somatic mutations being the consequence of the *POLE* hypermutator tumor phenotype [12, 20].

Regarding the prevalent mutations reported up to now, *POLE* p.L424V has been detected in 24 independent families [5, 11–15, 17], whereas *POLD1* p.S478N has been found in 4 independent families [5, 14]. Additionally, new rare variants located in the active exonuclease domain of these two proteins have also been reported. Among them, alteration of the proofreading activity as evidence of their pathogenicity was confirmed for some variants by functional assays in yeast, T4 bacteriophage or *E. coli* orthologous polymerases. A functional validation of the exonuclease activity was previously available for *POLE* p.D368V, p.Y458F [14, 18], and *POLD1* p.D316H, p.D316G, p.P327L and p.L474P [5, 11, 15], or it was specifically produced for *POLE* p.W347C [19].

Finally, the phenotype associated to this new hereditary CRC syndrome is not well defined yet and a better definition of the clinical characteristics will most likely help to detect potential mutation carriers in the general population. Accordingly, the aim of our study was to screen the exonuclease domain of *POLE* and *POLD1* in 155 patients with multiple polyps and early-onset CRC in order to find mutations affecting the replication fidelity of these proteins and shed light on this matter. Our final goal was to facilitate genetic counseling in order to correctly implement preventive strategies in those families.

## RESULTS

### Patient characteristics

Three subgroups of patients were studied including those presenting multiple polyps, early-onset CRC or MMR-defective CRC without germline alterations in the known hereditary CRC genes. Patients with multiple polyps presented 10–100 polyps, being the main precursor lesion adenomatous, serrated or a combination of adenomatous and serrated polyps with an age of onset < 70 and no alterations in the *APC* or *MUTYH* genes. Another selection criteria for the multiple polyps group included having at least one first-degree relative with multiple polyps, CRC, advanced adenomas or endometrial cancer diagnosed before the age of 70. Early-onset CRC patients were selected with an age of onset < 50 and no alterations in *MUTYH* or the MMR genes/proteins and tumor microsatellite stability. CRC patients with MMR deficiency presented loss of MMR protein (MLH1, MSH2, MSH6, PMS2) expression by immunohistochemistry with neither detected germline mutation in the MMR genes, nor somatic *MLH1* hypermethylation. Clinical characteristics are summarized in Table 1. Our patient cohort included predominantly cases with multiple polyps phenotype (83 cases, 53.5%), most of them presenting adenomas (67.5%) with a mean polyp onset age around 57 y.o. The early-onset CRC group corresponded to 59 cases (38.1%) and distal location of the tumor was predominant. No alterations in *APC* or *MUTYH* genes were present in the polyposis group, and alterations in *MUTYH* or the MMR genes/proteins and tumor microsatellite instability were absent in the early-onset CRC group. The smallest group (13 patients, 8.4%) was the MMR-defective CRC without germline alterations detected in the MMR genes. Noteworthy, adenomas were also commonly present in both selected CRC groups (59.3% and 66.7%).

**Table 1:** Clinical characteristics of the 155 patients with multiple polyps, early-onset CRC or mismatch repair-defective CRC

Multiple polyps patients		
Number of cases	83	
Females	26 (31.3%)	
*Type of lesion/polyps*		
AFAP*	56 (67.5%)	
SerratedP*	4 (4.8%)	
Mixed polyps*	6 (7.2%)	
Unknown^#^	17 (20.5%)	
Polyp onset, range (mean)	32–70 y.o. (56.8)	
Adenomas, range (mean)	4–91 (28)	
Serrated polyps, range (mean)	0–63 (7.8)	
CRC	21 (24.7%)	
Other neoplasms^^^	5	
Advanced adenoma	54	
FDR with CRC	50 (60.2%)	
FDR with endometrial cancer	9	
FDR with advanced adenoma	15	
**CRC patients**		
	**Early-onset CRC**	**MMR-defective CRC**
Number of cases	59	13
Females	33 (55.9%)	6 (46.15%)
CRC onset, range (mean)	22–50 y.o. (41.9)	31–68 y.o. (53.7)
*CRC location*		
Proximal to the splenic flexure	15 (25.4%)	7 (53.8%)
Distal	36 (61%)	3 (23.1%)
Unknown^#^	8 (13.5%)	3 (23.1 %)
Patients with adenomas	35 (59.3%)	8 (66.7%)
Adenomas, range (mean)	0-25 (3.1)	0-18 (4)
Patients with other neoplasms^	2 (3.3%)	2 (15.4%)
Familial CRC type X	11 (19.3%)	—
FDR with CRC	33 (57.9%)	1 (7.4%)
FDR with EC	4 (7%)	3 (25%)
FDR with AA	14 (24.6%)	1 (7.7%)

Abbreviations: AFAP, attenuated familial adenomatous polyposis; y.o., years old; CRC, colorectal cancer; FDR, first degree relative; MMR, mismatch repair; EC, endometrial cancer; AA, advanced adenoma.

*The main precursor lesions were 10–100 adenomatous, serrated or mixed polyps.

^#^Clinical data was partially available for 17 multiple polyposis and 3 MMR-defective CRC cases.

^^^Include those located in thyroid gland, bladder, prostate, pancreas, breast, uterus, lymphatic cells, kidney and larynx.

### Variant detection

After screening the exonuclease domain of *POLE* and *POLD1* in our cohort of patients, we detected several genetic variants that are listed in Table 2. Firstly, it is important to highlight that none of the two previously described recurrent mutations in *POLE* (p.Leu424Val) and *POLD1* (p.Ser478Asn) were present in our cohort. Among the detected variants, some of them corresponded to polymorphisms with an allelic frequency > 10%, they were also present in accessed human genetic variant databases with a similar frequency and they were not considered for further analysis. We proceeded to select those genetic variants with an allelic frequency < 10% or not present in any of the checked human genetic variant databases. Among them, some variants were located in intronic sequences or corresponded to coding synonymous variants. Their putative involvement in abnormal splicing processing was evaluated by using several bioinformatics tools. Results are summarized in Supplementary Table 1. None of the analyzed variants showed a prediction of altered splicing by 2 or more tools. However, one of the variants corresponded to a deletion of 71 base pairs in intron 13 of the *POLE* gene. This variant was not present in any of the human genetic variation databases but it was found in 2 early-onset CRC patients in our cohort, so we decided to further characterize it. In order to do so, we analysed its putative splicing alteration in a carrier at the RNA level by using RT-PCR. When compared to a control RNA not carrying this variant, we did not detect any additional amplification band in the deletion carrier being indicative of no splicing alteration, which was also confirmed by Sanger sequencing (Supplementary Figure 1).

**Table 2:** Genetic variants detected in screening of the exonuclease domain of the *POLE* and *POLD1* genes

*POLE*							
Variant	Location	rs ID	GMAF	EVS	ExAC	CSVS	Inner allelic frequency
c.910-157A > G	intronic	rs5744759	0.282	NA	NA	NA	0.374
c.910-6G > C	intronic	rs4077170	0.443	0.2957	0.6307	0.406	0.571
c.1020+29C > T	intronic	rs369332806	0.0002	NA	0.0001591	NA	0.0032
c.1020+46C > A	intronic	rs375701878	0.0002	NA	0.000135	NA	0.0032
c.1226+13G > A	intronic	rs577646338	NA	NA	0.0001171	NA	0.0032
c.1226+44G > A	intronic	rs79883120	0.0014	NA	0.0001274	NA	0.0097
c.1226+45C > T	intronic	rs5744761	0.0655	0.0464	0.05914	0.043	0.0161
c.1359+43G > A	intronic	rs4883555	0.4235	0.4302	0.4957	0.313	0.365
c.1359+46del71	intronic	NA	NA	NA	NA	NA	0.0065
c.1359+144G > T	intronic	rs5744776	0.0569	NA	NA	NA	0.0581
c.1420G > A (p.Val474Ile)	missense	NA	NA	NA	NA	NA	0.0032
*POLD1*							
Variant	Location	rs ID	GMAF	EVS	ExAC	CSVS	Inner allelic frequency
c.970 + 79G > A	intronic	rs559071730	0.0002	NA	NA	NA	0.0032
c.971 – 93G > C	intronic	NA	NA	NA	NA	NA	0.0032
c.1137 + 19C > G	intronic	rs572449832	0.0002	NA	NA	NA	0.0032
c.1137 + 53G > A	intronic	rs1673043	0.2925	NA	NA	0.054	0.0613
c.1137 + 69G > A	intronic	NA	NA	NA	NA	NA	0.0032
c.1138-8A > G	intronic	rs41544624	0.0002	0.001	0.0006018	0.004	0.0032
c.1173C > T (p.Asp391 =)	synonymous	rs2230244	0.0274	0.0003	0.008229	0.001	0.0032
c.1182C > T (p.Thr394 =)	synonymous	rs377462923	0.0002	0.0001	0.0001565	NA	0.0032
c.1485C > T (p.Thr495 =)	synonymous	rs2230245	0.077	0.1147	0.124	0.112	0.1419
c.1494 + 198T > A	intronic	NA	NA	NA	NA	NA	0.0032
c.1495 – 109A > C	intronic	rs3219395	0.011	NA	NA	NA	0.0226
c.1495 – 107C > T	intronic	NA	NA	NA	NA	NA	0.0032
c.1495 – 105C > T	intronic	rs559203182	0.0006	NA	NA	NA	0.0065
c.1495 – 44A > G	intronic	rs3219396	0.018	0.0212	0.02073	NA	0.029
c.1687 – 49G > A	intronic	rs368086982	NA	NA	NA	NA	0.0032
c.1687 – 38C > T	intronic	rs373705242	NA	0.0008	0.0007298	0.002	0.0032

Abbreviations: GMAF, global minor allele frequency; EVS, exome variant server; ExAC, exome aggregation consortium; NA, not available or not present in databases.

rs ID: variant identifier reported in dbSNP for this variant (http://www.ncbi.nlm.nih.gov/SNP/).

GMAF: Minor Allele Frequency in the 1000Genomes database (http://www.1000genomes.org/).

EVS: allelic frequency of the variant in European Americans in Exome Variant Server database (http://evs.gs.washington.edu/EVS/).

ExAC: allelic frequency of the variant in Exome aggregation consortium browser (http://exac.broadinstitute.org/).

CSVS: allelic frequency of the variant in CIBERER Spanish Variant Server (http://csvs.babelomics.org/).

Inner allelic frequency: Allelic frequency in our cohort of 155 patients (310 alleles).

### Novel *POLE* missense variant

**Figure 1:**
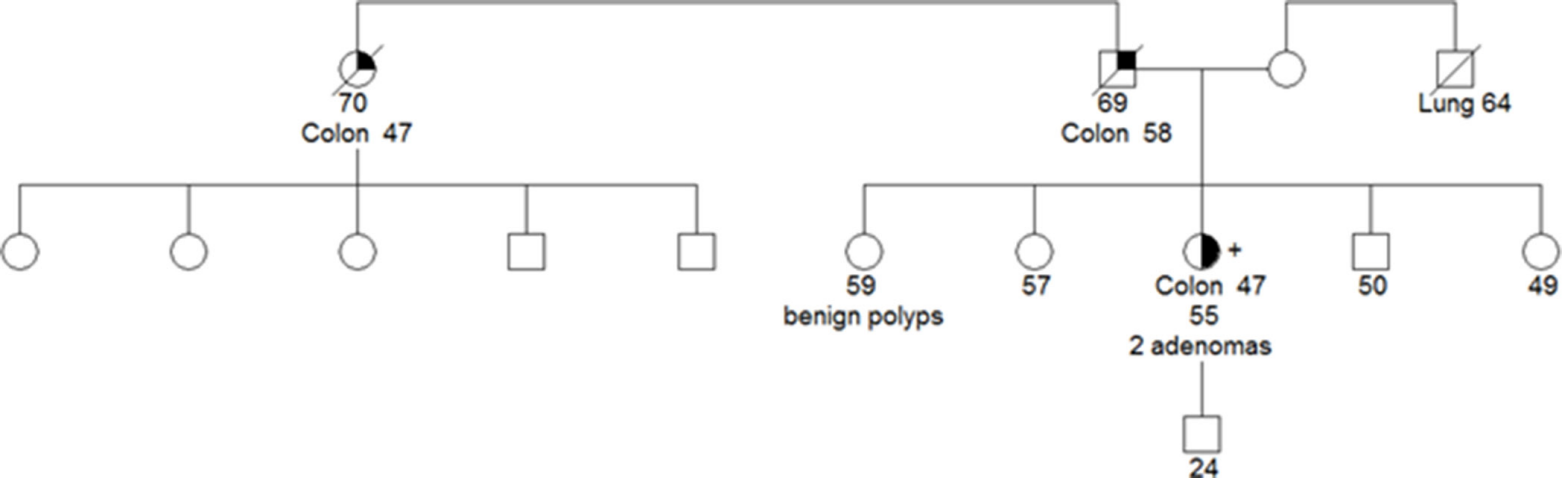
Pedigree for the proband carrying the *POLE* c.1420G > A (p.Val474Ile) variant

Instead of the previously described variants, we detected an interesting *POLE* missense variant in an early-onset CRC patient that corresponded to c.1420G > A (p.Val474Ile) (Table 2). The carrier proband presented a distal MMR-proficient CRC at the age of 47 and 2 non-advanced adenomas. The tumor showed expression of the MMR proteins, was stable and it did not present any histotype feature linked to MMR-defective tumors (tumor infiltrating lymphocytes, Crohn's like inflammatory reaction, mucinous, signet ring cells, medullary growth pattern). Her father and paternal aunt also had CRC at the age of 58 and 47, respectively, and one of her sisters presented non-advanced adenomas (Figure 1). This family fulfilled Amsterdam criteria and it could be considered an example of familial CRC type X. Unfortunately, variant segregation analysis was not feasible since DNA was unobtainable from additional family members.

**Figure 2:**
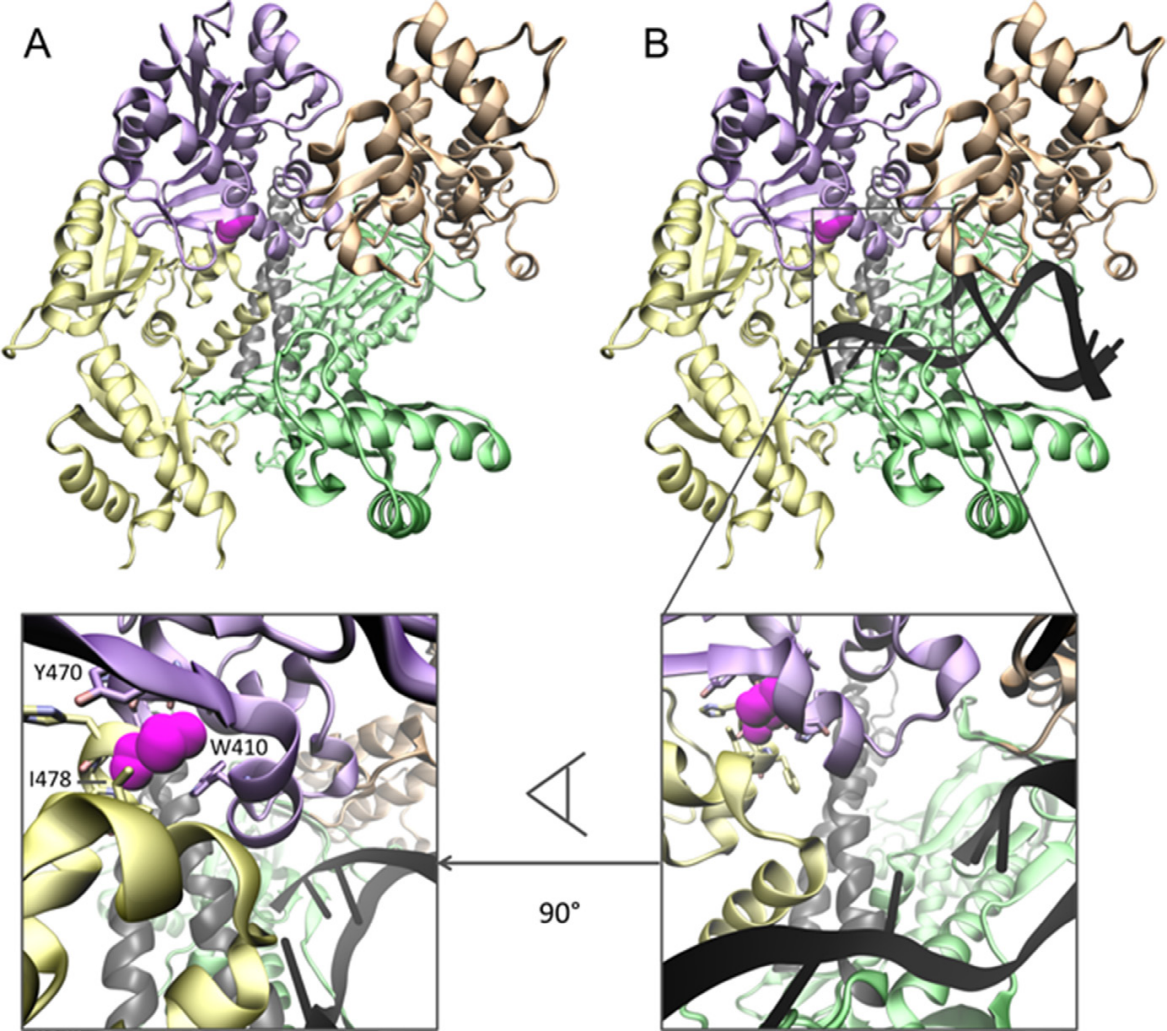
Structural analysis of the Val474Ile variant in the human POLE protein (**A**) Representation of the homology model. Polymerase domains are represented as follows: N-terminal (yellow), exonuclease (violet), palm (lime), thumb (orange) and fingers (silver). Position 474 is represented in magenta in van der Waals surface. (**B**) Superposition of the model and the template structure of the *Saccharomyces cerevisiae* DNA polymerase epsilon allows determining the relative position of the DNA chain (shown in black). A closer view to the region and its 90° rotation (lower inset) allow assessing the degree of proximity to DNA and the possible effects of the Val474Ile genetic variant on POLE domain packing.

The *POLE* c.1420G > A (p.Val474Ile) was suggestive of being a new putative pathogenic variant. It was not present in any of the accessed human genetic variation databases including a Spanish repository and the affected amino acid was found conserved in 100 vertebrates as well as in *D. melanogaster*, *C. elegans* and yeast. Regarding its protein location, Val474 is located in the N-terminal domain but very close to the C terminus end of the exonuclease domain (only 3 amino acids away). In order to assess the potential functional impact of the *POLE* Val474Ile variant, we analyzed further its position in the polymerase structure. To do so, we took advantage of the crystallized structure of the *Saccharomyces cerevisiae* DNA polymerase epsilon (PDB IB 4M8O). Indeed, the acceptable amount of sequence identity between human and yeast proteins (57%) had already been exploited for the construction of a homology model for human POLE (available at the ModBase database, UP Q07864). Analysis of this model allowed us to locate the Val474Ile variant in the N-terminal domain but in very close proximity to the exonuclease domain (Figure 2A). When superposed with the template structure (Figure 2B) along with DNA, it showed that the variant may not affect DNA binding directly. Conversely, it could have an indirect effect on the helical packing of the exonuclease and N-terminal domains, which could distort the physiological conformation needed for a correct polymerase activity. Furthermore, besides the structural analysis, additional bioinformatics assessment of this variant (using PolyPhen and CADD) predicted a deleterious effect for this amino acid change and most protein stability predictions were also in favor of a damaging effect (Eris, PoPMuSiC, I-Mutant).

### Functional studies in yeast for POLE Val474Ile

**Figure 3:**
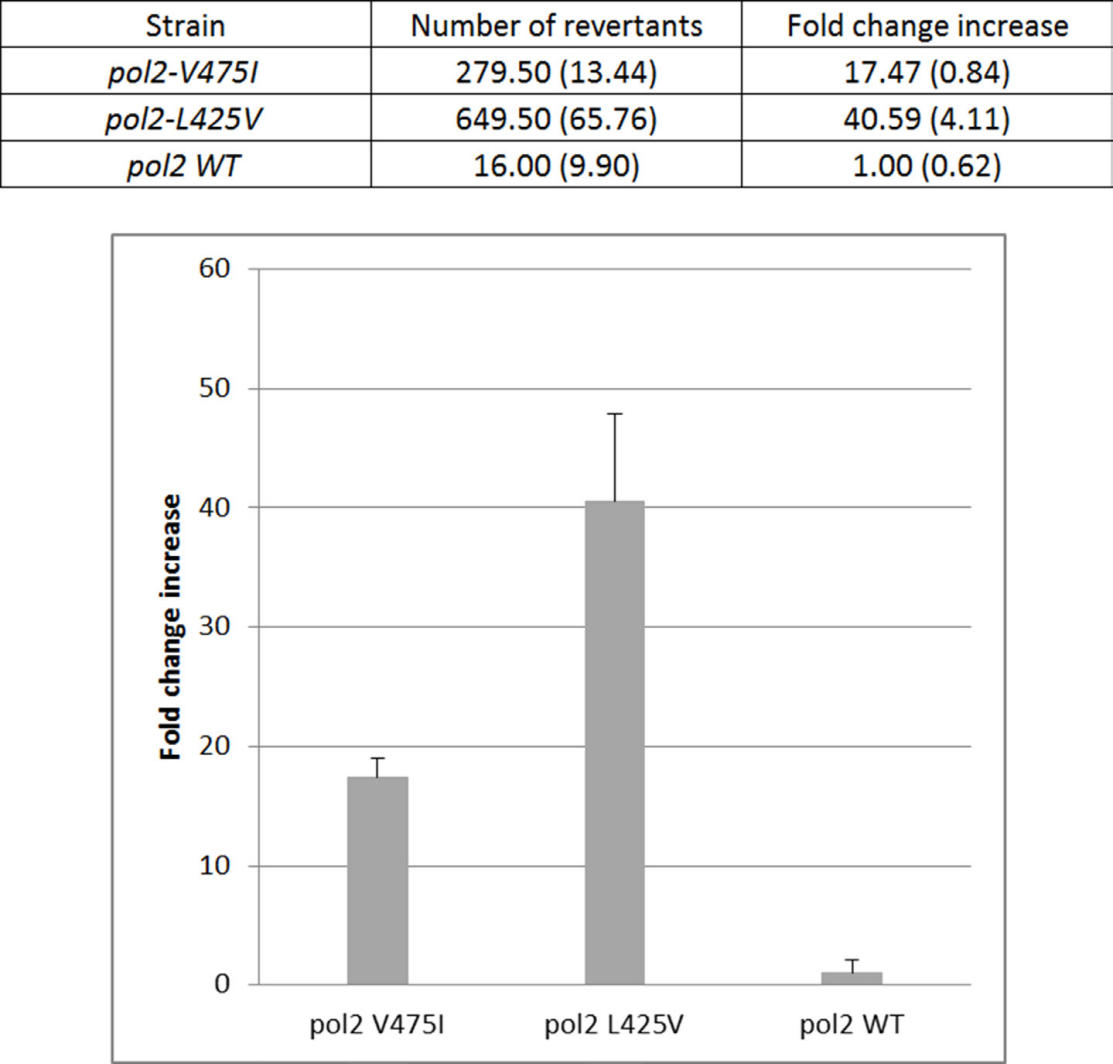
Mutation rates of *Schizosaccharomyces pombe* strains expressing pol2 p.L425V and p.V475I variants corresponding to human POLE p.L424V and p.V474I, respectively Mutation rates for Ade+ reversion of the *ade6*-485 allele are shown. Numbers were calculated from two experiments with two independently generated strains per 0,6 × 10^7^ cell divisions. In the graphic, error bars correspond to standard deviation (SD) of the fold-change increase in the number of revertants.

In order to further test the putative functional effect of this variant, we proceeded to analyze it in yeast. *POLE* V474 residue is highly conserved in eukaryotes, including *Schizosaccharomyces pombe* (*S. pombe*). We constructed the equivalent substitution in this organism, *pol2* p.V475I, and another strain carrying the equivalent change to the previously reported *POLE* p.L424V mutation (*pol2* p.L425V) as a positive control. We compared the *ade6*-485 allele reversion rates in these mutant strains with the wild-type *pol2* strand as negative control (Figure 3). When comparing *Pol2-L425V* substitution with the wild-type strain, 40 times more revertants were observed (*P-value* = 0.0108). Regarding *Pol2* p.V475I, the mutation rate was 17 times higher when compared to the wild-type strain (*P-value* = 0.0040).

### Somatic studies for *POLE* Val474Ile

Paired-tumor tissue was only available from the patient carrying the *POLE* p.Val474Ile variant. When tested on the corresponding tumor, loss of heterozygosity (LOH) could not be detected by Sanger sequencing in the carrier when comparing to her germline DNA. Whole-exome sequencing (WES) was also performed in the tumor DNA of the patient carrying the *POLE* p.Val474Ile variant to study the number and spectrum of somatic mutations. A second mutational event in the *POLE* gene was not found. We had available WES data obtained from four other MMR proficient CRC tumors that did not present germline or tumor alterations in *POLE* or *POLD1* to compare their number of substitutions and mutational spectrum with our *POLE* mutant. Regarding tumor WES results in the five samples analyzed, mean coverage was > 90× and > 79% of DNA in each tumor was sequenced with ≥ 30× coverage. First, a tumor profile for each sample was generated by eliminating variants present in a germline exome dataset. Mutation density plots suggested that tumour profiles were correctly generated and germline variants were mostly eliminated (Supplementary Figure 2). Additionally, WES data was normalized by selecting only those sequenced regions with coverage ≥ 10× in all five tumor samples. By doing so, a slight increase of the total number of substitutions in the tumor DNA from the p.Val474Ile germline carrier was detected when compared with the *POLE* wild-type MMR proficient tumors (Supplementary Table 2). However, when mutation spectrum was analyzed, the tumor DNA from the p.Val474Ile germline carrier did not show an increase in G > T/C > A or C > T/G > A transversions as suggested by previous studies.

## DISCUSSION

Our molecular screening of the *POLE* and *POLD1* exonuclease domains in a cohort of 155 patients with multiple polyps or early-onset CRC identified a novel *POLE* mutation in one family. We also detected several intronic variants most likely polymorphic and without pathogenic involvement.

Importantly, we did not find any of the previously described mutations for *POLE* (p.Leu424Val) and *POLD1* (p.Ser478Asn) in our cohort. As reported by previous studies, the *POLE* p.Leu424Val mutation frequency in multiple colorectal adenomas or familial CRC cohorts seems to be typically ≤ 0.3% [5, 11, 12, 14, 15], whereas the *POLD1* p.Ser478Asn mutation seems to be even less frequent (< 0.1%) [5, 14]. Taking our results into account, we can conclude that these mutations have indeed a very low frequency. On the other hand, we can also hypothesize that our sample size was probably not large enough to be able to detect any carrier of these mutations (334 samples needed to be screened to detect one carrier at a 0.3% frequency). Additionally, it could be also possible that the frequency of these mutations may be even lower in the Spanish population since only one carrier for the *POLE* p.Leu424Val mutation and no *POLD1* p.Ser478Asn mutation carriers have been reported so far [11].

Leaving aside these potentially recurrent mutations, other different variants have already been reported in *POLE* and *POLD1*. Most of them are located in the protein exonuclease domain and include p.Trp347Cys, p.Asn363Lys, p.Asp386Val, p.Lys425Arg, p.Pro436Ser, p.Tyr458Phe in *POLE* [13, 14, 17–20], and p.Asp316His, p.Asp316Gly, p.Pro327Leu, p.Arg409Trp, p.Leu474Pro for *POLD1* [5, 11, 15]. These previously reported variants and our newly identified variant are indicative that the entire coding region for *POLE* and *POLD1* should be screened instead of focusing only in a few variants.

It should also be noted that the phenotype selection criteria in our screened cohort included multiple polyps with at least one affected first-degree relative, early-onset CRC or MMR-defective CRC without germline alterations in the known genes. Previous studies have either used similar [11] or more permissive selection criteria [12–14] with similar results. It could be argued that including only multiple polyps with family history may have reduced the chances of detecting carriers. Considering previously reported known *POLE* or *POLD1* mutation carriers, the phenotypic spectrum included multiple polyps and early-onset CRC, as well as family history and would reinforce therefore the phenotype selection criteria used in our cohort. Regarding our molecular screening approach, we used PCR amplification of genomic DNA and subsequent Sanger sequencing corresponding to the entire exonuclease domain and adjacent intronic sequences. This approach is not biased to detect only *POLE* p.Leu424Val and *POLD1* p.Ser478Asn mutations as it was the case for some previous studies [11, 12] and permitted to detect additional mutations located in this region.

We were able to detect a new mutation in the *POLE* gene corresponding to c.1420G > A (p.Val474Ile). The heterozygous carrier was recruited in the early-onset CRC group and belonged to an Amsterdam I family without alterations in the MMR repair system. Its rarity, amino acid species conservation and location in the POLE protein already predicted a plausible pathogenic role. Our results in yeast suggest that the human *POLE* p.L424V and *POLE* p.V474I variants will cause an increased mutation rate due to faulty proofreading activity of this protein, although *POLE* p.V474I with an attenuated phenotype when compared to *POLE* p.L424V. However, since *POLE* Val474Ile is having a smaller effect on proof reading, other additional genetic variants also contributing to CRC predisposition cannot be disregarded.

Exome sequencing of the tumor corresponding to the p.V474I variant carrier revealed a slightly higher number of substitutions compared with four *POLE* wild-type tumors, although the increment was not as high as could be expected taking into account previously reported data [5, 10]. In this sense, when comparing with POLE-exonuclease mutant CRC tumor samples present in the COSMIC database (e.g. TCGA-CA-6718-01, P286R, 5,946 substitutions; and TCGA-A6-6141-01, p.S297F, 1,981 substitutions), a hypermutated profile was not evident. However, it should be noted that our tumor exome data normalization and filtering strategy was more stringent and probably some somatic mutations were overlooked. Besides, the percentage of G > T/C > A transversions was not increased compared with the other *POLE* wild-type tumors.

It is also worth mentioning that the same variant was previously reported as somatic in the COSMIC database in a gastric cancer with microsatellite instability (TCGA-BR-6452-01) [24]. This gastric tumor presented, as defined by the authors, “an ultramutated profile”, with 11,375 substitutions, and a mutation rate of approximately 283 mutations per Megabase. The percentage of G > T/C > A transversions in this sample was 10.29%. Notably, this ultramutated tumor presented three other somatic missense variants in *POLE* located far away from the exonuclease domain (p.R1111Q, p.S681R and Y1889C) that could be promoting even a stronger effect. In this regard, it is also worth mentioning that previous reports regarding *POLE* tumor mutation profile were generated mostly by using gene panel sequencing and with much higher coverage than the one used in the present study. We can hypothesize that our tumor profiling may have failed to show a clear distinctive profile for our novel *POLE* mutation due to a suboptimal filtering of germline variants and a limited sequencing coverage, as well as a milder mutator effect as shown by the functional assays in yeast.

In conclusion, we detected a new plausible *POLE* mutation, p.V474I, in an early-onset CRC patient. This variant is located right next to the exonuclease domain and affects protein function, leading to a proofreading activity defect as shown by yeast studies. The pathogenicity of this change was also suggested by bioinformatics and protein structure predictions. When checking its tumor profile, it showed an increase in the number of variants but not as strong as in the p.L424V POLE mutation in agreement with the yeast functional results. It is also worth mentioning that this is the first study to functionally analyse a *POLE* genetic variant outside the exonuclease domain in *POLE* and widens the spectrum of genetic changes in this DNA polymerase that could lead to CRC predisposition.

## MATERIALS AND METHODS

### Patients

Three subgroups of patients were studied including those presenting multiple polyps, early-onset CRC or MMR-defective CRC without germline alterations in the known hereditary CRC genes. Multiple polyps patients presented 10–100 polyps, being the main precursor lesion adenomatous, serrated or a combination of adenomatous and serrated polyps with an age of onset < 70 and no alterations in the *APC* or *MUTYH* genes. Another selection criteria for the multiple polyps group included having at least one first-degree relative with multiple polyps, CRC, advanced adenomas or endometrial cancer diagnosed before the age of 70. Early-onset CRC patients were selected with an age of onset < 50 and no alterations in *MUTYH* or the MMR genes/proteins and tumor microsatellite stability. CRC patients with MMR deficiency presented loss of MMR protein (MLH1, MSH2, MSH6, PMS2) expression by immunohistochemistry with neither detected germline mutation in the MMR genes, nor somatic *MLH1* hypermethylation.

We selected 155 patients (83 multiple polyps, 59 early-onset CRC and 13 MMR-defective CRC) from seven Spanish hospitals (Hospital Clínic de Barcelona, 61 patients; Complexo Hospitalario de Ourense, 28 patients; Fundación Pública Galega de Medicina Xenómica, Santiago de Compostela, 18 patients; Corporació Sanitaria Parc Taulí, 18 patients; Hospital Mútua de Terrasa, 14 patients; Hospital Vall d'Hebrón, 13 patients; Hospital de Donostia, 3 patients). This project respected the fundamental principles established in the Helsinki Declaration, including any later update, by the World Medical Association, by the European Council declaration related to human rights and biomedicine, in the UNESCO Universal Declaration about the human genome and human rights, as well as fulfilling the terms established by the Spanish legislation in the area of biomedical research, personal data protection and bioethics, and especially the Biomedicine Research law and Biobanking regulations (14/2007 3rd July and RD 1716/2011). This study was approved by the institutional ethics committee of each participating hospital and written informed consent was obtained at diagnosis on a systematic basis.

Germline DNA samples used for Sanger sequencing were obtained from peripheral blood, whereas in one case showing a relevant POLE variant, formalin-fixed, paraffin-embedded (FFPE) tumor DNA was also isolated for LOH and tumor profiling studies using the QIAamp DNA Blood Kit or QIAamp Tissue Kit, respectively (QIAGEN, Redwood City, USA) and following manufacturers' instructions. RNA for splicing analysis was obtained from peripheral blood collected in a PAXgene Blood RNA tube in one patient and isolated using the PAXgene blood RNA kit (PreAnalytiX, Hombrechtikon, Switzerland) following the manufacturer's protocol.

### Variant detection

The entire exonuclease domain of *POLE* and *POLD1* was screened for mutations by PCR amplification using custom primers designed with Primer3Plus (Supplementary Table 3) and subsequent Sanger sequencing (GATC Biotech, Germany). For the *POLE* gene, 2,095 nucleotides were sequenced including exons 9–14 and covering protein residues 267–491. The sequenced region for the *POLD1* gene covered 2,538 nucleotides, included exons 8-13 and comprised protein residues 304-562. Besides including the coding sequence for the entire exonuclease domain of *POLE* and *POLD1*, additional flanking intronic sequences were also screened, including introns 9, 11 and 13 entirely and fragments of introns 8, 10, 12 and 14 for *POLE*, as well as introns 7, 8, 9 and 11 entirely and introns 10, 12 and 13 partially for *POLD1*. Resulting sequences were compared to the human reference genome hg19 and visualized using Chromas (http://technelysium.com.au/) and ClustalW2 (http://www.ebi.ac.uk/Tools/msa/clustalw2/) All variants that differed from the reference genome were reported and their frequency was checked in several human genetic variation databases including dbSNP (http://www.ncbi.nlm.nih.gov/SNP/), 1000Genomes (http://www.1000genomes.org/), Exome Variant Server (http://evs.gs.washington.edu/EVS/), Exome Aggregation Consortium (http://exac.broadinstitute.org/) and CIBERER Spanish variant server (http://csvs.babelomics.org/).

### Variant evaluation

Synonymous and intronic variants with an allelic frequency < 10% were analyzed with Human Splicing Finder, (http://www.umd.be/HSF/) Berkeley Drosophila Genome Project (http://www.fruitfly.org/seq_tools/splice.html) and SPANR (http://tools.genes.toronto.edu/) in order to predict plausible splice site alterations.

One rare intronic variant in the *POLE* gene was studied at the RNA level by RT-PCR and PCR amplification using custom primers located two exons upstream and downstream from the variant (Supplementary Table 3) and Sanger sequencing to verify for correct exon splicing. A nonsynonymous variant with an allelic frequency < 10% was evaluated with bioinformatics tools (Polyphen, http://genetics.bwh.harvard.edu/pph2/ and CADD_phred, http://cadd.gs.washington.edu/score) in order to predict its possible effect on the protein function. This variant has been submitted to the ClinVar database (http://www.ncbi.nlm.nih.gov/clinvar/; accession number SUB1552845). Amino acid conservation in 100 vertebrates was also checked in the UCSC genome browser (http://genome.ucsc.edu/).

### Structural analysis

The sequence of DNA polymerase epsilon catalytic subunit A (POLE) was retrieved from Uniprot (ID Q07864) and used to perform a protein blast against the Protein Data Bank. That revealed that the closest available crystal structure was the *Saccharomyces cerevisiae* DNA polymerase epsilon (PDB IB 4M8O, http://www.rcsb.org/pdb/explore/explore.do?structureId=4M8O) [25]. Indeed, a model of human POLE (identity of 57% with 4M8O) is deposited in the ModBase database (annotated as UP Q07864, http://modbase.compbio.ucsf.edu/modbase-cgi/index.cgi) [26]. This model was visualized using the VMD 1.9.1 software [27] and the Stamp plugin was used to superpose the model with the original template to assess the relative position of DNA. Protein stability predictions were performed using Eris (http://troll.med.unc.edu/eris/), PoPMuSiC (http://dezyme.com/en/Software), I-Mutant 2.0 (http://folding.biofold.org/i-mutant/i-mutant2.0.html) and CUPSAT (http://cupsat.tu-bs.de/).

### Functional assays

Both *pol2*-L425V and *pol2*-V475I *S. pombe* mutant strains (encoding variants equivalent to human *POLE* L424V and V474I respectively) were constructed by cloning the wild type *pol2* gene fragment into the pFA6a-kanMX6 vector, followed by point mutation and insertion into an *ade6*-485 strain by recombination. The *pol2* wild-type segment was amplified using primers Pol2 fw (including *BglI*I restriction site) and Pol2 rev (including *Asc*I restriction site) (Supplementary Table 3). The PCR product and the pFA6a-*kanMX6* plasmid were digested with *Asc*I/*Bgl*II and *Asc*I/*Bam*HI respectively, and *Bgl*I and *Bam*HI sites were abolished after ligation. Point mutations were performed with Quikchange lightning site-directed mutagenesis kit (Agilent, Santa Clara, CA, USA) using primers Pol2-L425V fw + Pol2-L425V rev for *pol2*-L425V and Pol2-V475I fw + Pol2-V475I rev for *pol2*-V475I (Supplementary Table 3). The created pFA6a-*kanMX6* plasmids carrying wild-type *pol2* version, *pol2*-L425V or *pol2*-V475I mutants were integrated into *pol2* locus of the ade6-485 strain after linearization with *Bam*HI and selecting for G418/Geneticin resistance. Constructs were verified by Sanger sequencing. Mutation rates of the ade6-485 allele in the different strain backgrounds (wild-type pol2 as negative control, pol2-L425V positive control and pol2-V475I as the assayed genetic variant) were determined by a modified version of the previously described fluctuation analysis [28]. Two colonies were used for each construct. Cultured cells were distributed in 10 plates per construct (0.6 × 10^7^ cells/plate). Experiments were performed twice. Plates were scored after 12 days and the Student's *t-test* was used to calculate the statistical significance of the different results for the three constructs.

### Somatic analysis

LOH was studied in FFPE tumor DNA comparing to the germline DNA of a patient carrying a missense variant in *POLE* by PCR amplification using specific primers in the region surrounding the variant designed (Supplementary Table 3) and subsequent Sanger sequencing (GATC Biotech, Germany).

WES was performed in the tumour DNA of a patient carrying a missense variant detected in *POLE* and four other MMR proficient CRC tumors that did not present germline or tumor alterations in *POLE* or *POLD1* in order to study the number and spectrum of somatic mutations. WES was characterized by using the HiSeq2000 platform (Illumina, San Diego, USA) and SureSelectXT Human All Exon for exon enrichment V4 (Agilent, Santa Clara, USA). Since paired germline WES data was not available for the analyzed samples and to select only for tumor-only variants, we first selected only heterozygous substitutions not present in a germline WES dataset of 65 individuals to generate a somatic profile for the five samples analyzed. Results took into account only those regions with coverage > 10× in all samples in order to normalize for DNA and sequencing quality.

## SUPPLEMENTARY MATERIALS FIGURES AND TABLES


