# Supplementary material for: POLE and POLD1 screening in 155 patients with multiple polyps and early-onset colorectal cancer
Source: Oncotarget. 2017 Mar 1;8(16):26732–43. doi: 10.18632/oncotarget.15810 (PMC5432293; doi:10.18632/oncotarget.15810)
Supplement: Supplementary file 1 [file oncotarget-08-26732-s001.pdf]

POLE and *POLD1* screening in 155 patients with multiple polyps and early-onset colorectal cancer

Supplementary Materials

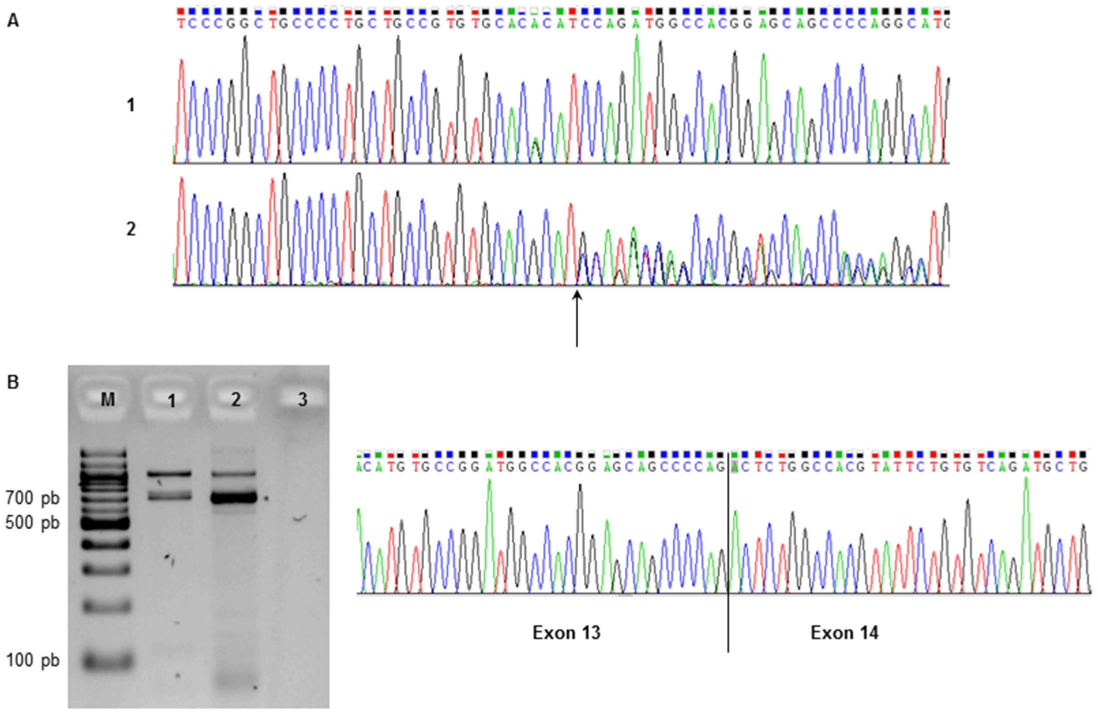

Supplementary Figure 1: DNA and RNA analysis of the c.1359+46del71 variant in POLE intron 13. (**A**) Sequencing result for POLE-3 PCR fragment in a control sample (1) and a carrier (2). The arrow indicates the presence of the deletion. (**B**) RNA analysis using the POLE DEL primers located in exon 11 and exon 16 in a control sample (1), a carrier (2) and negative control (3). The main PCR amplification corresponds to the expected size (697 base pairs). Sequencing of the main band yielded no alterations and showed intact exons 12 and 13 in the carrier as shown. An additional band of a bigger size was also present in both control and carrier corresponded to an alternative splicing including intron 15. (M) 100-base pair molecular weight marker.

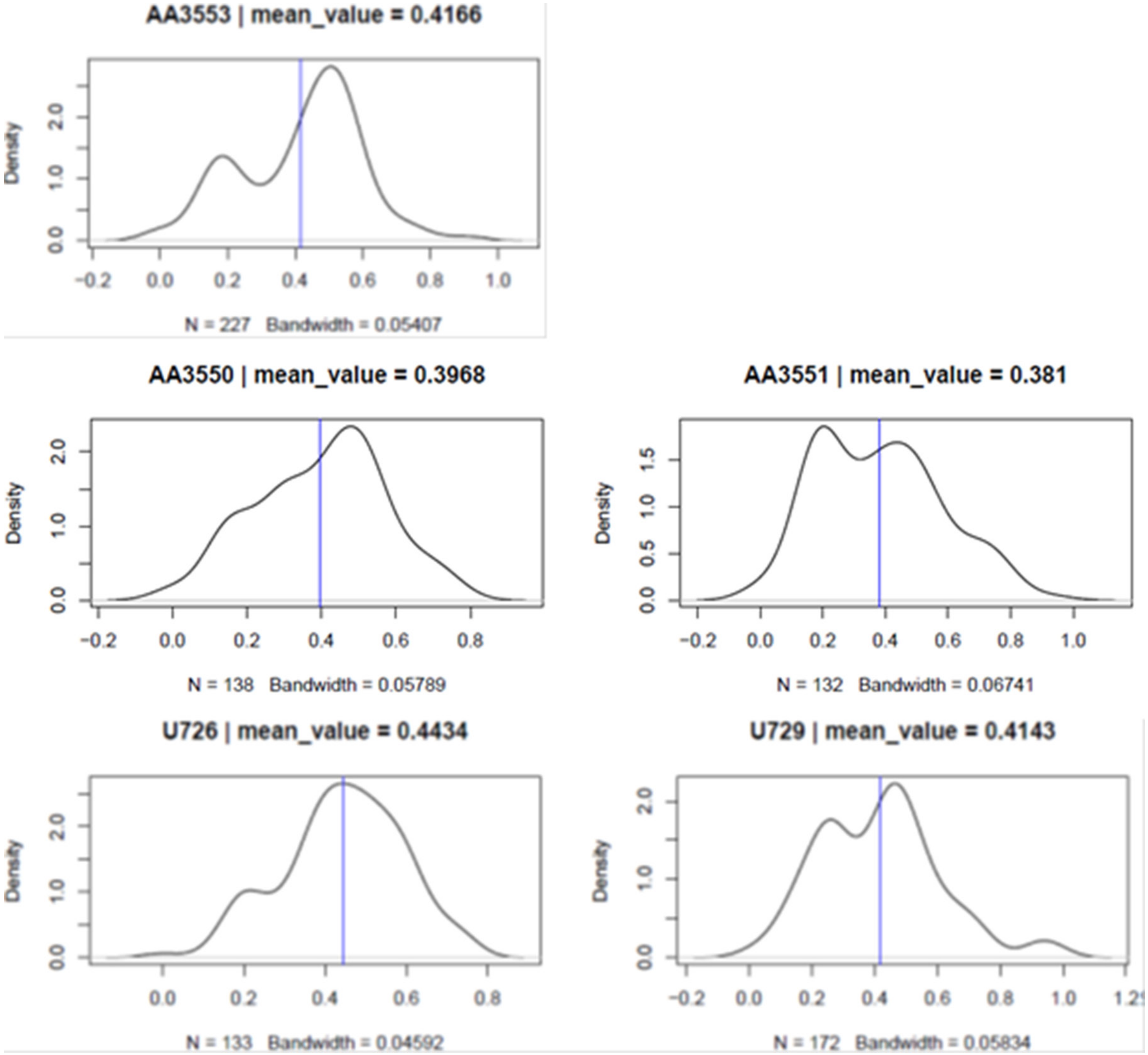

Supplementary Figure 2: Density plots for variants detected by WES in the five FFPE tumor DNA samples analyzed after filtering those present in a database of 65 germline exomes and normalizing by selecting only those sequenced regions with coverage above 10X in all samples. The X axis shows the proportion of all reads that present the alternative allele for all variants. Mean value has usually a proportion around 0.4 in tumor tissue (when tumor purity is 80%), whereas it corresponds 0.5 in germline tissue. These results will be in agreement of an appropriate germline filtering in our tumor dataset.

Supplementary Table 1: Rare intronic genetic variants tested for their putative involvement in abnormal splicing by using several bioinformatics tools

Gene	Variant	HSF	BDGP	SPANR
POLE	c.1020 + 29C > T	no effect (0% variation)	no effect (0% variation)	no effect (exon11 dPSI -0.21, exon10 dPSI 0.63)
POLE	c.1020 + 46C > A	no effect (0% variation)	no effect (0% variation)	slight effect (exon11 dPSI 0.14, exon10 dPSI-5.73)
POLE	c.1226 + 13G > A	no effect (0% variation)	NA	no effect (exon12 dPSI 2.60)
POLE	c.1226 + 44G > A	no effect (0% variation)	no effect (0% variation)	no effect (exon 12 dPSI -0.56)
POLE	c.1226 + 45C > T	no effect (0% variation)	NA	no effect (exon12 dPSI 2.85)
POLE	c.1359 + 46del71	no effect (0% variation)	no effect (0% variation)	NA
POLE	c.1359 + 144G > T	no effect (0% variation)	no effect (0% variation)	no effect (exon13 dPSI 2.49)
POLD1	c.970 + 79G > A	no effect (0.23% variation)	no effect (0% variation)	no effect (exon 8 dPSI -0.67, exon 9 dPSI 0.11)
POLD1	c.971 – 93G > C	no effect (0% variation)	no effect (0% variation)	no effect (exon 8 dPSI -0.55, exon 9 dPSI -0.12)
POLD1	c.1137 + 19C > G	no effect (0% variation)	no effect (0% variation)	no effect (exon 9 dPSI -0.14, exon 10 dPSI 0.07)
POLD1	c.1137 + 69G > A	no effect (0% variation)	no effect (0% variation)	no effect (exon 9 dPSI 0.14, exon 10 dPSI -0.36)
POLD1	c.1138 – 8A > G	no effect (0.1 %variation)	no effect (0% variation)	no effect (exon12 dPSI 0.07)
POLD1	c.1173C > T; (p.Asp391 =)	no effect (0% variation)	no effect (0% variation)	no effect (exon10 dPSI -0.30)
POLD1	c.1182C > T; (p.Thr394 =)	no effect (0% variation)	no effect (0% variation)	no effect (exon10 dPSI -0.20)
POLD1	c.1485C > T; (p.Thr495 =)	no effect (0.7 % variation)	no effect (0% variation)	no effect (exon12 dPSI 1.87)
POLD1	c.1494 + 198T > A	no effect (0% variation)	no effect (0% variation)	no effect (exon 12 dPSI -0.17, exon 13 dPSI 0.21)
POLD1	c.1495 – 109A > C	no effect (2.2% variation)	no effect (3% variation)	no effect (exon12 dPSI 0.17)
POLD1	c.1495 – 107C > T	no effect (0% variation)	no effect (0% variation)	no effect (exon13 dPSI -0.20)
POLD1	c.1495 – 105C > T	no effect (0.97%variation)	no effect (4% variation)	no effect (exon13 dPSI -0.20)
POLD1	c.1495 – 44A > G	no effect (0% variation)	no effect (0% variation)	no effect (exon13 dPSI -1.88)
POLD1	c.1687 – 49G > A	no effect (0% variation)	no effect (0% variation)	no effect (exon13 dPSI -2.44, exon14 dPSI -1.90)
POLD1	c.1687 – 38C > T	no effect (0% variation)	no effect (0% variation)	no effect (exon13 dPSI-0.84, exon14 dPSI 0.20)

HSF, Human Splicing Finder; BDGP, Berkeley Drosophila Genome Project; SPANR, Splicing-based Analysis of Variants; dPSI, maximum difference across tissues in percentage of transcripts with the exon spliced; NA, not available.
Human Splicing Finder: Variants affecting splicing have a 10% of variation [21].
BDGP: Berkeley Drosophila Genome Project. Variants considered to affect splicing have a percentage of variation of 10% [22].
SPANR: Variants predicted to affect splicing have a dPSI above 5 or below -5 [23].

Supplementary Table S2: Normalized number of heterozygous variants not present in a germline exome dataset that were found in FFPE tumor DNA from p.Val474Ile variant carrier (AA3553) and four colorectal tumor samples without alterations in *POLE* or the MMR system (AA3550, AA3551, U726, U729)

Number of variants	AA3553 (*POLE* p.Val474Ile)	AA3550 (*POLE* WT)	AA3551 (*POLE* WT)	U726 (*POLE* WT)	U729 (*POLE* WT)
C>T	47	30	22	26	24
G>A	50	32	34	31	41
C>T / G>A	97	62	56	57	65
A>G	22	13	18	15	23
T>C	31	17	13	18	23
A>G / T>C	51	30	31	33	46
C>G	6	8	7	10	14
G>C	10	7	4	8	7
C>G / G>C	16	15	11	18	21
G>T	11	3	4	2	6
C>A	5	3	6	4	11
G>T / C>A	16	6	10	6	17
A>C	10	12	6	4	6
T>G	6	2	0	3	6
A>C / T>G	16	14	6	7	12
A>T	12	7	7	8	5
T>A	17	4	11	4	6
A>T / T>A	29	11	18	12	11
Total number of substitutions	227	138	132	133	172

Mutation spectrum (%)	AA3553 (*POLE* p.Val474Ile)	AA3550 (*POLE* WT)	AA3551 (*POLE* WT)	U726 (*POLE* WT)	U729 (*POLE* WT)
C>T / G>A	42.73	44.93	42.42	42.86	37.79
A>G / T>C	23.35	21.74	23.48	24.81	26.74
C>G / G>C	7.05	10.87	8.33	13.53	12.21
G>T / C>A	7.05	4.35	7.58	4.51	9.88
A>C / T>G	7.05	10.14	4.55	5.26	6.98
A>T / T>A	12.78	7.97	13.64	9.02	6.40

The normalization was applied counting only those variants situated in genomic regions with coverage above 10× in all samples. Mutation spectrum is expressed in percentages of each type of substitution divided by the total number of substitutions. WT = wild-type.

Supplementary Table 3: Custom DNA oligonucleotides

Sequencing primers for the exonuclease domain of *POLE* and *POLD1*

GENE	FRAGMENT	Forward primer sequence 5′-3′	Reverse primer sequence 5′-3′	PCR size (bp)
POLE	POLE-1	ATGGGGAGTTTAGAGCTTGG	ACGGTCATACCCTGAGAACA	736
	POLE-2	CCATGAGCTTTGTTCTCAGG	CTGCCATACTCTTGGGTGAC	738
	POLE-3	TCCTGATGAAGGGTTTTCAC	CCGACAGGACAGATAATGCT	742
POLD1	POLD1-1	CTCACTTCTCCGGCCTCTAT	TTCTCGTAGCTCTGCACCTT	814
	POLD1-2	CCTGTCATCCAGATCTGCTC	GCCTCTGATAAAAGCCACAG	718
	POLD1-3	CTCAATCTCCGTTCTTCAGG	ACAGAGGTCACAGGAGTGGA	602
	POLD1-4	CCACGTCTGACCTCACTCTT	GAGAAGGTGGGAAATGGAGT	716

Primers for LOH analysis in FFPE tumor DNA of the p.V474I variant carrier

GENE	FRAGMENT	Forward primer sequence 5′-3′	Reverse primer sequence 5′-3′	PCR size (bp)
POLE	POLE-3 FFPE	TCTGTGCTTCACACTTGACC	CCGACAGGACAGATAATGCT	206

Primers for cDNA sequencing in POLE (exons 11–16)

GENE	FRAGMENT	Forward primer sequence 5′-3′	Reverse primer sequence 5’-3’	PCR size (bp)
POLE	POLE DEL	CATCATGGTCACCTACAACG	AGGCACTTTCTCCTCTTCCT	697

Primers for functional assessment in *S.pombe*

GENE	FRAGMENT	Forward primer sequence 5′-3′	Reverse primer sequence 5′-3′	PCR size (bp)
Pol2	POL2 WT	CGCGCGAGATCTCTGATTCGTT<br>TAAGCTTTTTCAGTTAATGGTGG	CTAAAGGCGCGCCGGTACAT<br>TGAGGCGACATCAAGATGG	2593
	POL2-L425V	GGGTAAAGAGAGATAGTTATT<br>TACCTCAAGGAAGTCAAGGTGT<br>CAAAGCTGTCACTGTCA<br>GTAAATTAGGTTATAATCC	GGATTATAACCTAATTTACTGACAGT<br>GACAGCTTTGACACCTTGACTTCCTT<br>GAGGTAAATAACTATCTCTCTTTACCC	2593
	POL2-V475I	TTTCAGATGCTGTTGCTACTTAT<br>TTTCTTTACATGAAATATATCCAT<br>CCTTTCATTTTTTCTCTTTGTAA<br>CATTATCCC	GGGATAATGTTACAAAGAGAAAAAA<br>TGAAAGGATGGATATATTTCATGTAAA<br>GAAAATAAGTAGCAACAGCATCTGAAA	2593

bp, base pairs.
